# Role of *Staphylococcus aureus* Virulence Factors in Inducing Inflammation and Vascular Permeability in a Mouse Model of Bacterial Endophthalmitis

**DOI:** 10.1371/journal.pone.0128423

**Published:** 2015-06-08

**Authors:** Ajay Kumar, Ashok Kumar

**Affiliations:** 1 Department of Ophthalmology, Kresge Eye Institute, Wayne State University, Detroit, Michigan, United States of America; 2 Department of Anatomy and Cell Biology, Wayne State University, Detroit, Michigan, United States of America; 3 Department of Immunology and Microbiology, Wayne State University, Detroit, Michigan, United States of America; UC Irvine Medical Center, UNITED STATES

## Abstract

*Staphylococcus (S*.*) aureus* is a common causative agent of bacterial endophthalmitis, a vision threatening complication of eye surgeries. The relative contribution of *S*. *aureus *virulence factors in the pathogenesis of endophthalmitis remains unclear. Here, we comprehensively analyzed the development of intraocular inflammation, vascular permeability, and the loss of retinal function in C57BL/6 mouse eyes, challenged with live *S*. *aureus*, heat-killed *S*. *aureus *(HKSA), peptidoglycan (PGN), lipoteichoic acid (LTA), staphylococcal protein A (SPA), α-toxin, and Toxic-shock syndrome toxin 1 (TSST1). Our data showed a dose-dependent (range 0.01 μg/eye to 1.0 μg/eye) increase in the levels of inflammatory mediators by all virulence factors. The cell wall components, particularly PGN and LTA, seem to induce higher levels of TNF-α, IL-6, KC, and MIP2, whereas the toxins induced IL-1β. Similarly, among the virulence factors, PGN induced higher PMN infiltration. The vascular permeability assay revealed significant leakage in eyes challenged with live SA (12-fold) and HKSA (7.3-fold), in comparison to other virulence factors (~2-fold) and controls. These changes coincided with retinal tissue damage, as evidenced by histological analysis. The electroretinogram (ERG) analysis revealed a significant decline in retinal function in eyes inoculated with live SA, followed by HKSA, SPA, and α-toxin. Together, these findings demonstrate the differential innate responses of the retina to *S*. *aureus* virulence factors, which contribute to intraocular inflammation and retinal function loss in endophthalmitis.

## Introduction

Infectious endophthalmitis is one of the most devastating complications of ophthalmic surgeries and penetrating injuries [1]. The most common isolated microorganisms are Gram-positive staphylococci, which constitute up to 90% of all bacterial pathogens [2]. The course of infectious endophthalmitis varies widely depending upon the organism involved, ranging from therapeutically responsive infections to therapeutically challenging infections caused by more virulent pathogens such as *Bacillus cereus* [3, 4] and *Staphylococcus(S) aureus* [5–7]. As one of the most feared ocular pathogens, *S*. *aureus* causes severe intraocular inflammation, significant vision loss, and can even cause loss of the eye [8, 9]. Despite therapeutic and surgical interventions, endophthalmitis results in partial or complete visual loss within a few days of microbial inoculation [10].

The current treatment for bacterial endophthalmitis involves intravitreal administration of antibiotics [11]. Some of the antibiotics, in the process of destroying the bacteria, release lipoteichoic acid (LTA) and peptidoglycan (PGN) from the bacterial cell walls, thereby exacerbating the acute inflammatory response [12, 13]. Indeed, previous studies have shown that the Gram-positive bacterial cell wall can induce cytokine production, inflammatory cell chemotaxis, and cellular toxicity in a number of experimental models, including endophthalmitis [14, 15]. Similarly, our previous studies have implicated the role of Toll-like receptors (TLRs) in mediating retinal innate responses to *S*. *aureus* cell wall components, including PGN and LTA [16–18]. In addition to cell wall components, *S*. *aureus* produces various toxins, such as α-toxin and Toxic-shock syndrome toxin (TSST1). However, their role in eliciting retinal innate responses remains elusive [6, 19].

The pathogenesis of bacterial endophthalmitis involves complex host-pathogen interactions that results in intraocular inflammation, vascular leakage, and retinal tissue damage. The relative contribution of *S*. *aureus* virulence factors in evoking these innate responses is not well understood. Thus, in the current study, we investigated the role of individual virulence factors in the pathogenesis of staphylococcal endophthalmitis and comparisons were made with live and heat-inactivated *S*. *aureus*. Together, our data suggest that *S*. *aureus* virulence factors incite differential innate responses in the retina and suggest that the neutralization of a single, specific virulence factors may not be effective in preventing/treating bacterial endophthalmitis.

## Material and Methods

### Ethics Statement

Female C57BL/6 (aged ~8 weeks) specific pathogen free mice obtained from the Jackson Laboratory were maintained at the Kresge Eye Institute in specific pathogen free conditions. All the procedures were conducted in compliance with the ARVO statement for the Use of Animals in Ophthalmic and Vision Research, and were approved by the Institutional Animal Care and Use Committee of Wayne State University (protocol A-08-02-13).

### Bacterial strain and virulence factors

**Fig 1:**
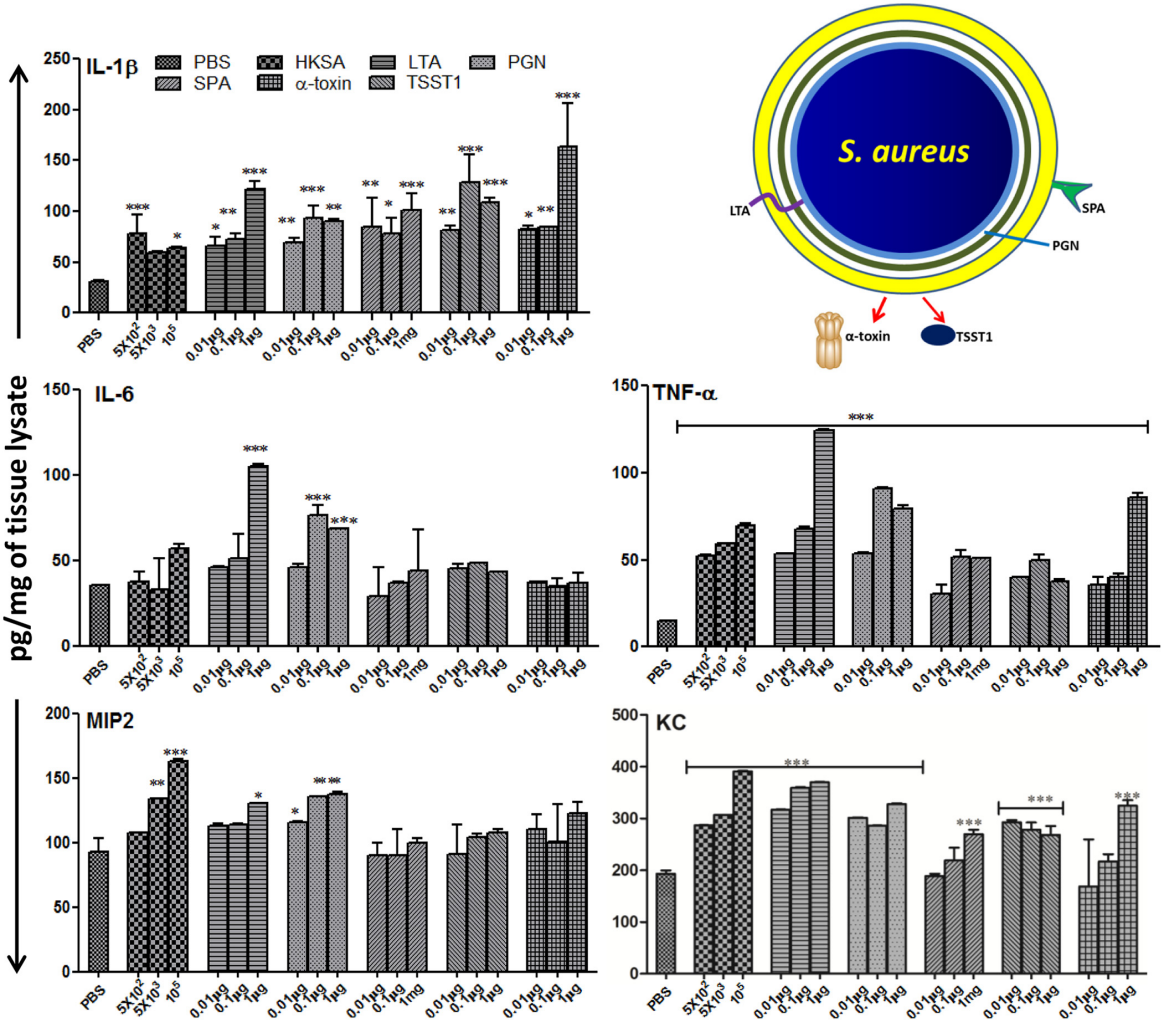
Effect of *S*. *aureus* virulence factors on inflammatory responses. Eyes of C57BL/6 mice (4–6 per group) were inoculated with indicated dose of heat-killed *S*. *aureus* (HKSA) (5X10^5^ CFU/eye), its cell wall components (PGN and LTA; 0.1μg each), and cell surface and secreted proteins (SPA, TSST, and α-toxin; 0.1μg each). After 24h, eyes (n = 6) were enucleated and subjected to ELISA, eyes injected with PBS served as controls. Statistical analysis was performed by using one way ANOVA with Dunnett’s multi-comparison test. *p <0.05, **p <0.005, ***p<0.0005.

The *S*. *aureus* strain RN6390 was used to induce endophthalmitis [20, 21]. The bacterial strain was maintained and grown in tryptic soy broth (Sigma Aldrich, St. Louise, USA) overnight at 37°C. The bacterial count was adjusted to 5000 cfu/ml in PBS. For the preparation of heat killed *S*. *aureus* (HKSA), 10^5^ cfu/ml of bacterial culture was boiled in a water bath for 10 min., followed by a viability assay using bacterial plating. Purified PGN, SPA, α-toxin, TSST1, and LTA from *Staphylococcus aureus* were purchased from Sigma Aldrich, USA. A dose response study was performed to select the suitable dose that worked for each bacterial virulence factor to elicit inflammation (Fig 1). Alpha-toxin was tested for hemolytic activity in 5% sheep blood agar before injection. All the virulence factors were dissolved in endotoxin-free water and checked for endotoxin levels prior to injection by using LIMULUS amoebocyte lysate assay (Genescript, NJ, USA). The endotoxin levels in LTA, PGN and TSST1 were <0.005 EU/μg while in α-toxin and SPA it was <0.05 EU/μg, of protein.

### Induction of endophthalmitis

C57BL/6 mice were maintained in a 12 h light/dark cycle and temperature was controlled at 22°C. Mice were provided free access to the water and standard laboratory chow. During experiment, mice were anesthetized by intraperitoneal injection of ketamin/xylazine (ketamin, 100–125 mg/kg; xylazine, 10–12.5 mg/kg). For intravitreal injections, a 32-G needle attached to a 10 μl glass syringe (Hamilton, Reno, USA) was used under a dissecting microscope. Mice were injected with live *S*. *aureus* (5000 CFU), heat-killed *S*. *aureus* (HKSA), or bacterial factors as indicated in 1.

### Enzyme-linked immunosorbent assay (ELISA)

Following injection with either *S*. *aureus* or bacterial virulence factors, the mouse eyes were enucleated and crushed in a tissue lyser and protein was estimated using a protein estimation kit (Thermoscientific, USA) according to manufacturer’s instructions. To determine the levels of cytokines and chemokines in the retinal tissue lysates, ELISA was performed using ELISA kits for TNF-α, IL-1β, and IL-6 (BD biosciences, San Diego, CA, USA) and MIP2 and KC (R & D systems, Minneapolis, MN, USA) according to manufacturer’s instructions. All of the values were expressed as mean ± standard deviations (SD).

### Real-time PCR

The expression of MMP2, MMP3, MMP13, S100A7, and S100A9 were determined with qRT-PCR from mice treated with virulence factors. The retinas were removed and processed for ultrasonication on ice. Total RNA was extracted using TRIzol solution (Invitrogen, Carlsbad, CA, USA) according to the manufacturer’s Instructions. cDNA was synthesized using 2 μg of total RNA by reverse transcription (Thermo scientific, Rockford, IL, USA). Sybergreen based qRT-PCR was performed using specific primers, as listed in Table 1. GAPDH was used as a housekeeping gene to calculate the relative quantification. qRT-PCR was performed using the StepOnePlus real-time PCR system (Applied Biosystem, Grand Island, NY, USA). The data analysis was performed using the 2^−ΔΔ^ *CT* method.

**Table 1:** Sequences and product sizes of PCR primers.

**Gene ID**	**Primer sequences**	**Product size (bp)**
**MMP2**	F: CCGATCTACACCTACACCAAGAAC	107
	R: CCAGTACCAGTGTCAGTATCAG	
**MMP9**	F: CTCTACAGAGTCTTTGAGTCCG	143
	R: CCTGTAATGGGCTTCCTCTATG	
**MMP13**	F: CTGGACCAAACTATGGTGGG	135
	R: GGTCCTTGGAGTGATCCAGA	
**S100A7**	F: TGCACCAAGAGCAACAGACT	229
	R: CCATGAAGCGAGGCACACTA	
**S100A9**	F: GCTCCTCGGCTTTGACAGAGTGCAAC	92
	R: GCATTTGTGTCCAGGTCCTCCATGAT	

### Neutrophil infiltration

Flowcytometry was used to determine the extent of PMN infiltration in the retina, as described earlier [18]. The retinas were removed from mice injected with bacterial virulence factors, HKSA, or live *S*. *aureus* and digested with Accumax (Millipore, Billerica, MA, USA) for 10 min. at 37°C. Retinal tissue was passed from a 23-G needle/syringe to make a single cell suspension and filtered through a 40-μm cell strainer (BD Falcon, San Jose, CA, USA). Blocking was performed by using Fc block for 30 minutes and then washing with PBS containing 0.5% BSA. Cells were incubated in the dark at room temperature with conjugated monocloncal antibodies and respective isotypes for 30 minutes. Again, washing was performed in PBS with 0.5% BSA and cells were acquired on a BD Accuri C6 (BD Immunocytometry Systems, San Jose, CA, USA). The data analysis was performed using Flow Jo (Tree Star, Inc., Ashland, OR, USA).

### Fundus imaging and fluorescein angiography

For fundoscopic examination, mice pupils were dilated with a mixed ophthalmic solution containing 0.5% atropine sulfate (E. Fougera & Co., NY, USA) and 1.25% phenylephrine hydrochloride (Wilson Ophthamic, OK, USA). The fundus was photographed with a Micron III (Phoenix Research Lab., Pleasanton, CA, USA) fundus camera for small animals. Mouse pupils were dilated, and mice were intraperitoneally injected with 20% AK-FLUOR (Akorn, Inc., Lake Forest, IL, USA) at a dose of 0.01 ml/5 g of mouse body weight. Photographs were taken with Micron III containing a barrier filter for fluorescein angiography and processed for Photoshop for digital images.

### Assessment of vascular permeability

Immunohistochemistry was performed on retinal tissue sections from mice injected with bacterial virulence factors, live *S*. *aureus*, or HKSA for the purpose of determining the extent of vascular permeability. CD31 (a vascular marker) and fibrinogen (a leakage marker) were used for the immunostaining. Eyes were enucleated and fixed in 4% para-formaldehyde for 2–3 hrs, followed by washing in PBS for 5 minutes. The eyes were placed in 5%, 10%, 20% sucrose in PBS for 30 minutes each, followed by 30% sucrose plus embedding medium (Tissue-Tek OCT compound, Sakura Fintek, USA Inc., Torrence, CA, USA) in a 1:1 ratio overnight at 4°C. The eyes were fixed in OCT, sectioned, and mounted on slides. For immunostaining, sections were kept at 4% para-formaldehyde for 15 minutes, and then washed twice in PBS for 5 minutes each. For permeabilization and to prevent non-specific binding, sections were incubated in 1% BSA containing 0.5% Triton-X100. Primary antibodies for CD31 (1:100, Abcam, Cambridge, MA, USA) and fibrinogen (1:20, Developmental Studies Hybridoma Bank, University of Iowa, Iowa City, IA.) diluted in blocking solution were allowed to sit overnight in a humidified chamber at 4°C; secondary antibodies for goat anti-mouse-FITC (1:100) and PE-Cy3 anti-hamster (BD Bioscience, San Jose, CA, USA) were also used. Photomicrographs were taken using fluorescence microscope. Fluorescent intensity was measured using imageJ software and calculated using the following formula [22].

Fluorescence intensity = Integrated Density—(Area of selected cell X Mean fluorescence of background readings).

### Histology

Eyes from the euthanized mice were enucleated 24h post-injection for histopathological examination and fixed in 10% formalin for 24h. The embedding, sectioning, and hematoxylin and eosin stain (H&E) staining were performed by Excalibur Pathology, Inc. (Oklahoma City, OK, USA).

### Electroretinography (ERG)

Scotopic electroretinography (ERG) was used to determine retinal function following *S*. *aureus* infection and injection of bacterial virulence factors. The mice (control and infected) were anesthetized 24h after injection. The temperature of the mice was maintained at 37°C using a heat pad. The pupils were dilated using a 1% tropicamide ophthalmic solution. ERGs were recorded following bilateral mydriasis and at least 12h of dark adaptation. Indifferent, silver-embedded thread eye electrodes (OcuscienceLLC, Kansas City, MO) were used to record the ERG. Reference needle electrodes (stainless steel subdermal electrodes) were placed in anterior scalp and a ground needle electrode was placed in the tail. ERG responses were acquired using an ERG system (OcuscienceLLC, Kansas City, MO) and analyzed using ERGVIEW 4.600V. Ganzfeld light stimulus was used to present ten 10 ms flashes, with light intensities increasing from 0.0001 to 100 cd-s/m^2^. The amplitudes of the a- and b-waves were recorded. The ERG a-wave amplitude was measured between the ERG baseline and the first negative peak, while the ERG b-wave amplitude was measured between the first negative peak and the first positive peak.

## Results

### *S*. *aureus* virulence factors evoke distinct inflammatory responses in mouse eyes

Intraocular inflammation is a hallmark of bacterial endophthalmitis [2] and peaks between 24 and 48h post bacterial inoculation [5, 23, 24]. To investigate the role of *S*. *aureus* virulence factors in generating inflammatory responses, the eyes of C57BL/6 mice were injected with various doses of *S*. *aureus* cell wall components and toxins for 24h. To this end, our data showed a dose-dependent induction of inflammatory mediators, as evidenced by increased levels of cytokines (IL-1β, TNF-α, and IL-6) and chemokines (MIP-2 and KC) by all virulence factors (Fig 1). However, the expression pattern was differentially regulated. For example, IL-6 and MIP2 levels were significantly higher in HKSA, PGN, and LTA injected eyes. In contrast, TSST1, α-toxin, and SPA seem to have no effect on these cytokines. The levels of TNF-α and KC were significantly increased by all virulence factors. Interestingly, IL-1β was induced more by the higher concentration of toxins. Overall, the cell wall components seem to induce higher inflammatory responses as compared to toxins.

**Fig 2:**
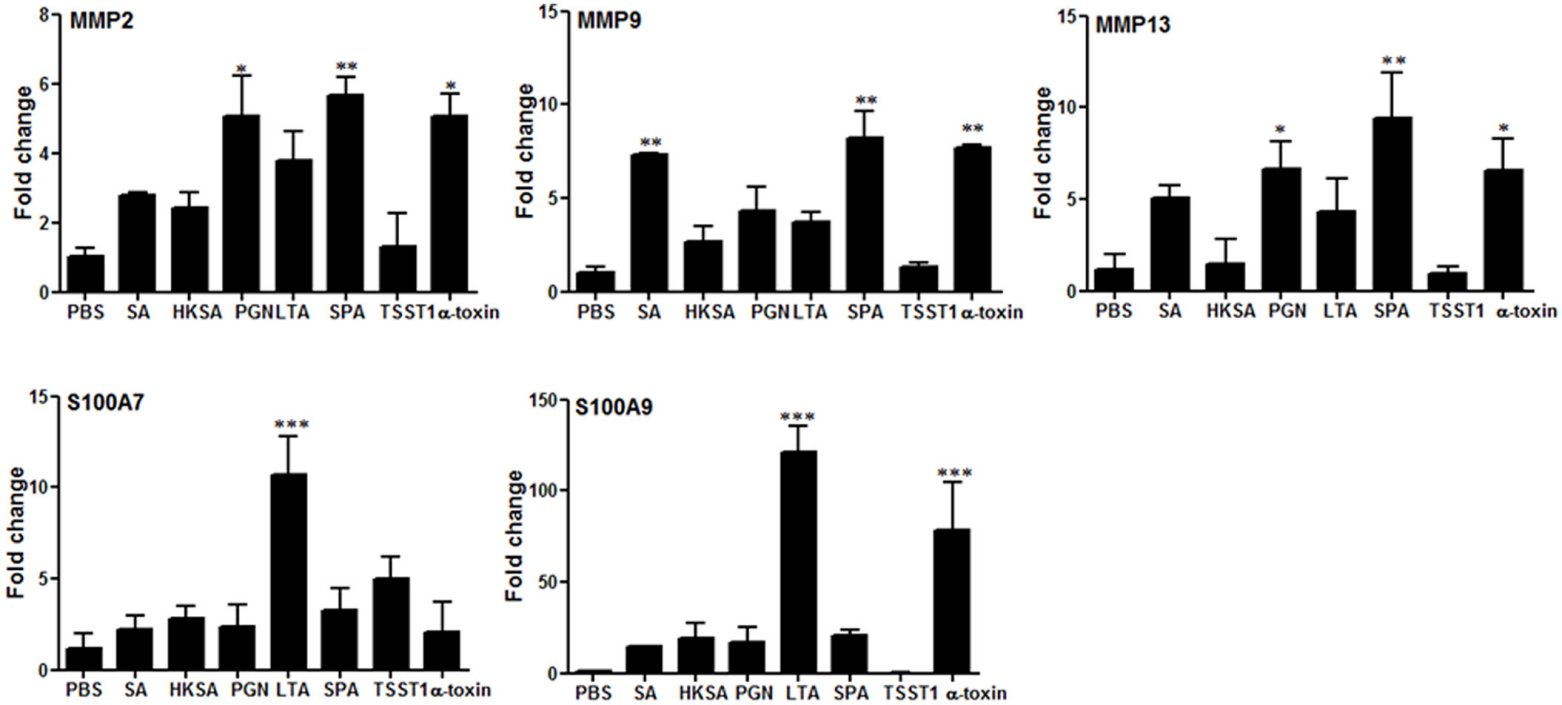
Expressions of MMPs and S100As in eye challenged with *S*. *aureus* and its virulence factors. Eyes of C57BL/6 mice (4–6 per group) were inoculated intravitreally with live *S*. *aureus* (5000 CFU/eye), HKSA (5X10^5^ CFU/eye), LTA (0.1μg/eye), PGN (0.1μg/eye), TSST1 (0.1μg/eye), α-toxin (0.1μg/eye), SPA (0.1μg/eye), and PBS (2μl) for 24h. The mRNA expression of MMP2, MMP9, MMP13, S100A7, and S100A9 was determined by Real-time RT-PCR. Statistical analysis was performed by using one way ANOVA with Dunnett’s multi-comparison test. *p <0.05, **p <0.005.

In addition to inflammatory mediators, we also assessed the expression of metalloproteinases (MMPs) and danger-associated molecular pattern (DAMPs, S100A7/S100A9), which are implicated in the inflammatory response. As shown in Fig 2, live *S*. *aureus* significantly induced MMP9 expression in the mouse retina, whereas MMP2, MMP13, S100A7 and S100A9 levels were increased, but did not reach statistical significance. In contrast, eyes injected with HKSA did not exhibit significant changes in any of the MMPs or DAMPs. Among the cell wall components, PGN stimulated the increased expression of MMP2 and MMP13, LTA-challenged eyes exhibited higher levels of S100A7 and S100A9, and SPA induced the expression of MMP2, MMP9, and MMP13. Among the toxins, α-toxin increased the expression of MMP2, MMP9, MMP13, and S100A9, while TSST-1 appears to have no effect on these mediators.

### Live *S*. *aureus* and PGN induced maximum PMN infiltration in the retina

**Fig 3:**
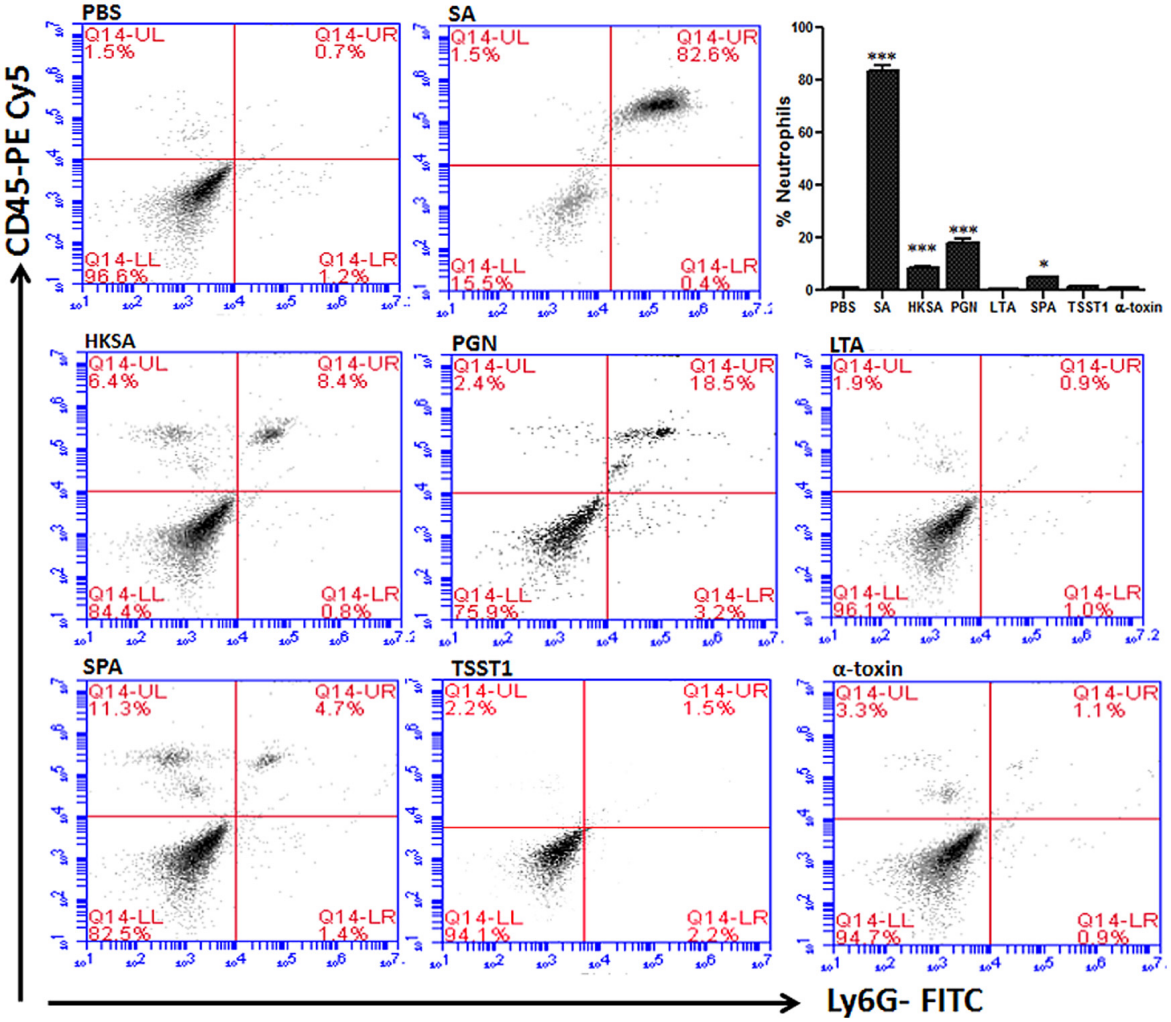
Effect of *S*. *aureus* virulence factors on neutrophil infiltration to the retina. Eyes of C57BL/6 mice (4–6 per group) were inoculated intravitreally with *S*. *aureus* (5000 CFU/eye), HKSA (5x10^5^ CFU), or and the indicated virulence factors (0.1μg /eye). At 24h post infection, eyes were enucleated and retinas from two eyes were pooled to make single-cell suspensions and stained with anti-CD45 and anti-Ly6G mAbs. Post-acquisition, the cells were size gated to differentiate them from debris. The percentage of dually positive PMNs was determined using a CD45 versus Ly6G dot plot (upper-right right [Q2] quadrant). The data are representative of duplicate experiments. Statistical analysis was performed by using one way ANOVA with Dunnett’s multi-comparison test. *p <0.05, ***p <0.0005.

Neutrophils are the first innate immune cells recruited to the retina in endophthalmitis [25]. To determine the effect of *S*. *aureus* virulence factors on PMN infiltration, we performed flowcytometry. As expected, the highest PMNs levels were observed in eyes challenged with live SA (82.6%), followed by PGN (18.5%), HKSA (8.4%), and SPA (4.7%) injected mice (Fig 3). Surprisingly, LTA was not found to induce significant PMN infiltration and a similar trend was observed in eyes challenged with toxins.

### *S*. *aureus* virulence factors increased vascular leakage and tissue damage in the retina

**Fig 4:**
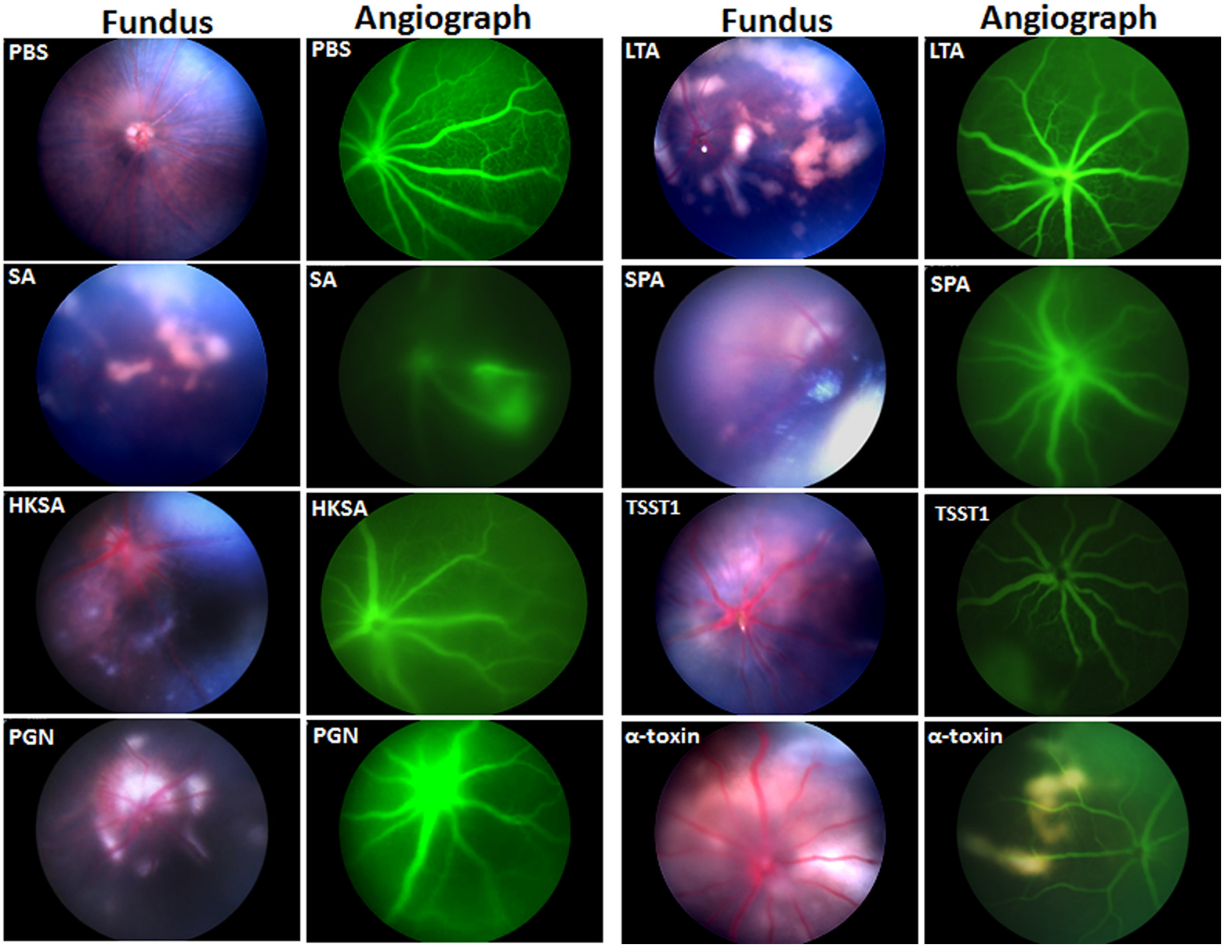
Fundus imaging of eyes challenged with *S*. *aureus* virulence factors. Eyes of C57BL/6 mice (4–6 per group) were inoculated intravitreally with *S*. *aureus* (5000 CFU/eye), HKSA (5x10^5^ CFU), or and the indicated virulence factors (0.1μg /eye). At 24h post infection, eyes were examined by fundus microscope and images were captured using Micron III. Angiography was performed by intraperitoneal injections of 2% fluorescent dye.

**Fig 5:**
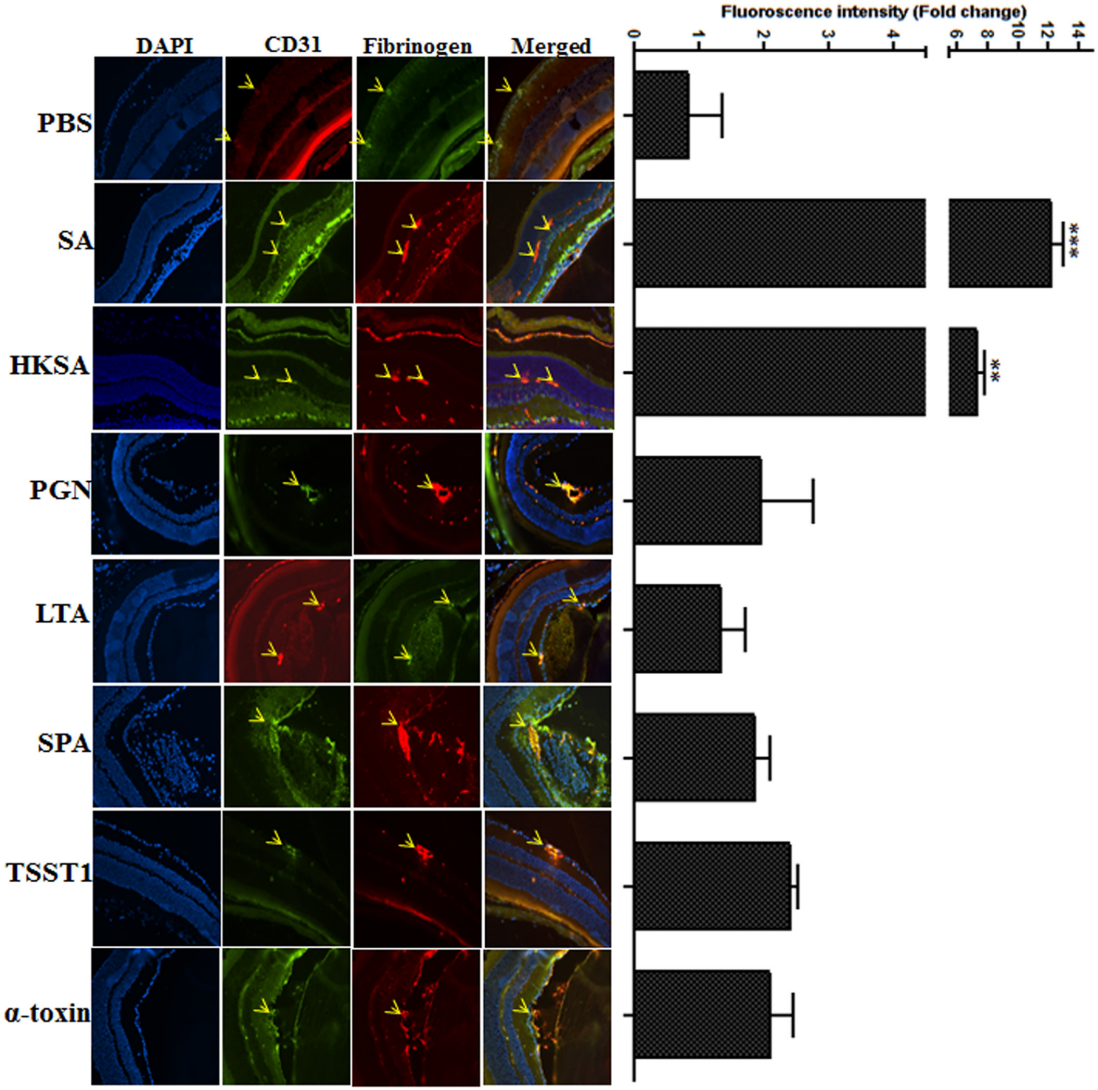
Determination of vascular leakage in infected retina. To determine the vascular permeability, infected eyes were embedded in OCT at 24h and the cryosections were subjected to IHC analysis. Fluorescent densities for CD31 (Green, biomarker for blood vessels) and Fibrinogen (Red, released from blood into the retinal tissue) were measured. Fluorescent intensities were scanned in three different regions of the retina in each slide and an average of 3 to 4 retina in each treatment group. Fold change was calculated by calculating the ratio between CD31 (Vascular marker) and Fibrinogen (Leakage marker) and presented as mean ± SD. Statistical significance was determined by One way ANOVA with Dunnett’s multi-comparison test. ***p<0.0005, **p<0.005, n = 6). Original magnification 20x.

The influx of PMNs and other immune cells to the retina is tightly controlled by the blood retinal barriers (BRB) [26, 27]. Funduscopic imaging combined with fluorescent angiography revealed retinal vessel occlusions, ischemia, and signs of vascular leakage in *S*. *aureus* virulence factor challenged eyes versus control (PBS) (Fig 4). To further confirm the breakdown of the BRB, we determined the degree of vascular permeability by assessing the release of fibrinogen using immunohistochemistry (Fig 5). Our data showed visible signs of vascular leakage in the retina of eyes injected with *S*. *aureus* virulence factors. The quantification of comparative fibrinogen intensity revealed significantly higher vascular leakages in the retina of *S*. *aureus* (12-fold) and HKSA (7.3-fold) injected mice, whereas eyes challenged with other virulence factors exhibited ≤ 2-fold increases.

**Fig 6:**
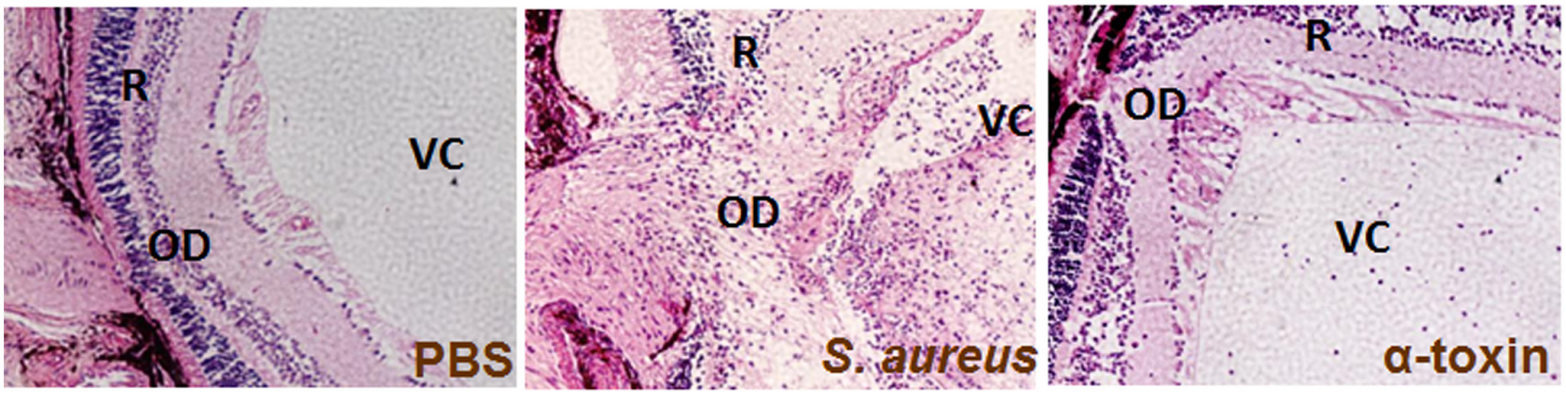
Histological assessment of the impact of *S*. *aureus* virulence factors on retinal tissue damage. Histological analysis was performed on eyes challenged with *S*. *aureus* virulence factors as indicated in Fig 2 legend. Representative images of eyes of inoculated challenged with *S*. *aureus* and α-toxin (0.1μg/eye) showed retinal damage. Original magnifications 20X. VC, Vitreous Chamber; R, Retina; OD, Optic Disk.

The effect of *S*. *aureus* virulence factors on retinal structural integrity was assessed using histological analysis (Fig 6). In control (PBS injected) eyes, the retinal structure was intact, with no significant changes. In contrast, eyes challenged with *S*. *aureus* exhibited severe retinal damage, as evidenced by a loss of architecture, retinal folding, edema, and the presence of infiltrated cells (Fig 6). Among the virulence factors, only α-toxin was found to cause mild retinal damage and edema.

### *S*. *aureus* virulence factors exerted a differential effect on retinal function

**Fig 7:**
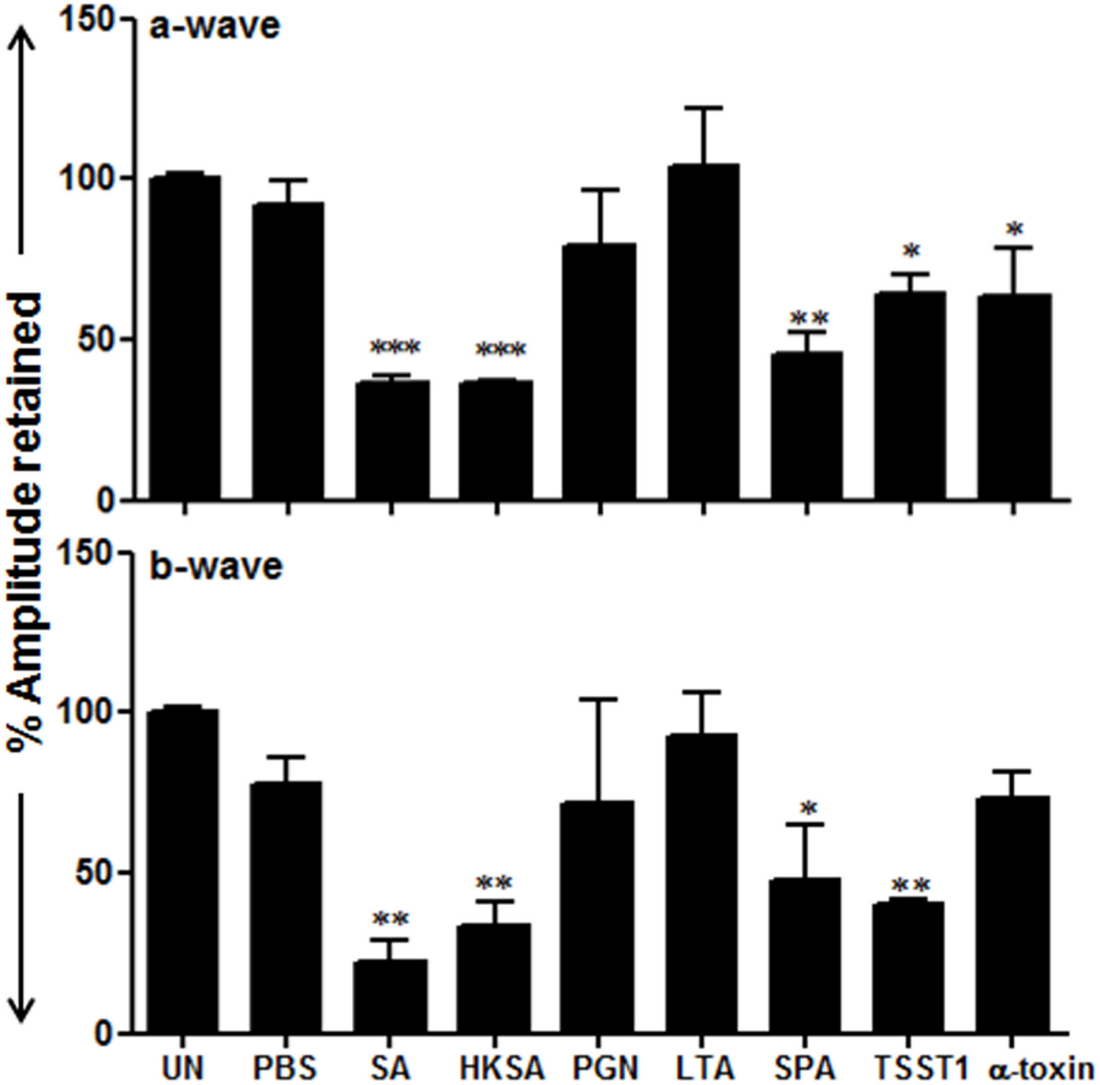
Retinal function analysis in *S*. *aureus* virulence factors challenged eyes. Eyes of C57BL/6 mice (4–6 per group) were inoculated intravitreally with *S*. *aureus* (5000CFU), HKSA (5x10^5^ CFU), LTA (0.1μg), PGN (0.1μg), SPA (0.1μg), TSST1 (0.1μg), α-toxin (0.1μg), and PBS (2μl). ERG was performed after overnight dark adaptation. Electroretinogram responses to a 6-dB flash were recorded and the percentage amplitude of a- and b-wave retained in infected eyes was compared to that of uninjected mice control and presented as mean ± SD. ERG amplitudes Statistical analysis was performed using one-way ANOVA with Dunnett’s multi-comparison test. *p <0.05, **p <0.005, ***p <0.001 (n = 6 mice were used per treatment). UN,uninjected.

To determine the impact of individual virulence factors on retinal function, ERG analysis was performed. As shown in Fig 7, both live *S*. *aureus* and HKSA caused a significant (~60–70%) decline in the amplitude of both a- and b-waves. Among the virulence factors, SPA and TSST1 reduced a- and b-wave amplitudes by 30–40%. PGN and LTA seem to have no significant impact of retinal function, whereas α-toxin causes decline in a-wave, but not b-wave, amplitude.

## Discussion

Previous studies from our laboratory and others have shown that *S*. *aureus* induces severe endophthalmitis in experimental models [7, 24, 28–31]. However, which virulence factors contribute to the induction of intraocular inflammation, BRB breakdown, and retinal function impairment, the hallmarks of endophthalmitis, remain unclear. In the present *in vivo* study, we evaluated the role of *S*. *aureus* cell wall components (HKSA, PGN, and LTA) and cell surface or secreted proteins (SPA, α-toxin, and TSST-1) on the pathogenesis of endophthalmitis. Our data showed that all virulence factors induced a concentration-dependent release of various inflammatory mediators (IL-1β, TNF-α, IL-6, MIP-2, and KC) in mouse eyes. These changes coincided with increased vascular leakage and PMN infiltration, resulting in diminished retinal function. Collectively, our study indicates that the pathogenicity of *S*. *aureus* is primarily due to the expression of a large variety of virulence factors, as the effect of any one virulence factors was not sufficient to cause endophthalmitis.

Similar to other organs/tissues, in the retina, the innate immune response prevent the establishment of an infection by recognizing pathogens at the early stages of infection and providing a first, rapid line of host defense [2, 16]. It is now well-established that the host uses pattern-recognition receptors (PRRs), such as TLRs, to recognize microbial-associated molecular patterns (MAMPs) present on pathogens [16]. In the case of Gram-positive bacteria, including staphylococci, PGN and teichoic acids (LTA and WTA) serve as MAMPs [32] and constitute 70% of the weight of their cell wall [33, 34]. Indeed, our *in vitro* studies have implicated the role of TLR2 in inducing the inflammatory response in retinal microglia following challenge with staphylococcal PGN and LTA [18]. Similarly, TLR2 was found to mediate the innate response of Müller glia towards *S*. *aureus* [35, 36]. We postulated that, under *in vivo* conditions, proliferating staphylococci could release cell wall components, which percolate through the vitreous and are recognized by retinal residential innate immune cells, such as the glial cells [17]. In the current study, we mimicked this situation by giving intravitreal injections of purified PGN and LTA and demonstrated that staphylococcal cell wall components induce the secretion of pro-inflammatory mediators and cause retinal vascular leakage. However, it is important to mention that the vascular leakage may not be due to the direct effects of PGN or LTA, but rather the secondary effects of intraocular inflammation.

In addition to their cell wall components, the damaging effects seen in staphylococcal infections could be due to an arsenal of virulence factors such as surface binding protein A (SPA) and secreted toxins [37]. Due to their IgG binding ability, the role of SPA has been primarily implicated in promoting immune evasion by inhibiting bacterial phagocytosis [38, 39]. However, studies from our laboratory [40] and others have shown that SPA exerts pro-inflammatory stimuli via tumor-necrosis factor receptor 1 (TNFR1) signaling [41, 42]. Here, we demonstrate that SPA induces the production of pro-inflammatory mediators, including TNF-α, suggesting its role in promoting intraocular inflammation in endophthalmitis. Staphylococcal superantigens, such as TSST1, manifest disease through hyper-activation of the immune system, resulting in massive cytokine production and septic shock [43]. Toxins also possess the ability to bind to human vascular epithelial cells and induce the production of pro-inflammatory mediators through the activation of NF-kB [44, 45]. *S*. *aureus* secreted α-toxin is a major cytolytic toxin which acts through inserting pores in the membrane of targeted host cells [46, 47]. The damaging effects of α-toxin have been demonstrated in staphylococcal endophthalmitis, as evidenced by the reduced pathogenicity of *Agr* and *Sar* mutants [6, 48]. In the current study, we demonstrated the ability of α-toxin to induce the inflammatory response, particularly via IL-1β, indicating another role of α-toxin in increasing the virulence of *S*. *aureus* in endophthalmitis. Previous studies have demonstrated the activation of the inflammasome by α-toxin as an underlying mechanism for IL-1β production [49]. Similarly, *S*. *aureus* mediated inflammasome activation has been reported in conjunctiva goblet cells [50]. Our preliminary studies (unpublished data) have also indicated the potential involvement of the inflammasome complex in staphylococcal endophthalmitis and studies are in progress to delineate the mechanisms.

The MMPs belong to the large family of zinc-dependent neutral endopeptidases that are capable of degrading the extracellular matrix. Although MMPs play an important role in injury repair during inflammation by cleaving components of extracellular matrix, the excessive expression of MMPs can also destroy the extracellular matrix and promote further inflammation. The role of MMP13 has been described in *Pseudomonas aeruginosa* induced corneal ulceration [51]. In diabetic retinopathy, the elevated expression of MMPs may facilitate an increase in vascular permeability via proteolytic degradation of the tight junction protein occludin, followed by disruption of tight junction complex [52]. Our data showed increased expression of MMP2, MMP9, and MMAP13, indicating their involvement in bacterial endophthalmitis. In addition to MMPs, increased levels of S100 proteins were also detected in *S*. *aureus*-infected retina. The S100/calgranulin complex has antimicrobial properties [47, 53] and is massively released by dying neutrophils to provide host defense [54]. Three main S100 proteins have been linked to innate immune function, as they are expressed in cells of myloid origin [55]. These S100 proteins can be secreted via an alternate route, bypassing the classic Golgi-rout, which is the typical mode of secretion for DAMP-related factors [56]. These DAMPs have a role in maintaining cellular homeostasis, but turn into pro-inflammatory danger signals when released into the extracellular environment following cell damage, infection, or inflammation [57].

The eye is protected from inflammatory cells due to the existence of blood retina barrier (BRB), which is composed of the retinal endothelium and retinal pigmented epithelial cells. Increased BRB permeability has previously been reported in bacterial endophthalmitis [26]. However, which bacterial virulence factors induce this response is not yet clearly defined. In the current study, we demonstrate increased vascular permeability, mainly in eyes injected with live *S*. *aureus*, indicating a synergistic effect of *S*. *aureus* virulence factors in inducing vascular permeability. Another mechanism of BRB breakdown could be due to increased levels of inflammatory mediators, as was demonstrated by increased PMN and mononuclear cell infiltration in rat eyes injected with IL-1β or TNF-α [58]. The combined effect of increased vascular permeability and intraocular inflammation culminates to impaired retinal function [5, 23, 59]. We hypothesize that some virulence factors, specifically toxins, could directly impact retinal function through their direct lytic action. Surprisingly, individual virulence factors were found to have little impact on retinal function, as evidenced by the observation that there was no significant decline in either a- or b-wave amplitude following injection.

## Conclusions

In this study, we comprehensively demonstrated the role *S*. *aureus* cell wall components and secreted virulence factors in evoking retinal innate responses leading to intraocular inflammation, vascular permeability, and a loss of retinal function. Although the ideal approach for the management of endophthalmitis should include both bacterial eradication and inflammation resolution, monotherapy with intravitreal antibiotic injections remains the current standard of treatment. The antibiotics, while destroying the bacteria, may release bacterial cell wall components, which contribute to intraocular inflammation in bacterial endophthalmitis. Thus, we need adopt both antimicrobial and adjunct anti-inflammatory therapeutic approaches to treat infectious endophthalmitis.

## Supporting Information

S1 ARRIVE Guideline Checklist Completed “The ARRIVE Guidelines Checklist” for reporting animal research experiments in this manuscript. (PDF)
